# Selective dorsal rhizotomy in non-ambulant children with cerebral palsy: a multi-center prospective study

**DOI:** 10.1007/s00381-023-06062-4

**Published:** 2023-07-13

**Authors:** Conor S. Gillespie, Benjamin J. Hall, Alan M. George, Dawn Hennigan, Christine Sneade, Stephanie Cawker, Adikarige Haritha Dulanka Silva, Michael Vloeberghs, Kristian Aquilina, Benedetta Pettorini

**Affiliations:** 1 Department of Neurosurgery, Alder Hey Children’s Hospital NHS Trust, Liverpool, UK; 2 Department of Clinical Neurosciences, University of Cambridge, Cambridge, UK; 3 Department of Neurology, Alder Hey Children’s Hospital NHS Trust, Liverpool, UK; 4 Department of Neurosurgery, Great Ormond Street Hospital for Children, London, UK; 5 Great Ormond Street Institute of Child Health, University College London, London, UK; 6 Department of Neurosurgery, Nottingham University Hospitals NHS Trust, Nottingham, UK

**Keywords:** SDR, Cerebral palsy, GMFCS, Multi-center

## Abstract

**Purpose:**

Assess the effects of selective dorsal rhizotomy (SDR) on motor function and quality of life in children with a Gross Motor Function Classification System (GMFCS) level of IV or V (non-ambulatory).

**Methods:**

This is a prospective, observational study in three tertiary neurosurgery units in England, UK, performing SDR on children aged 3–18 with spastic diplegic cerebral palsy, and a GMFCS level of IV or V, between 2012 and 2019. The primary outcome measure was the change in the 66-item Gross Motor Function Measure (GMFM-66) from baseline to 24 months after SDR, using a linear mixed effects model. Secondary outcomes included spasticity, bladder function, quality of life, and pain scores.

**Results:**

Between 2012 and 2019, 144 children who satisfied these inclusion criteria underwent SDR. The mean age was 8.2 years. Fifty-two percent were female. Mean GMFM-66 score was available in 77 patients (53.5%) and in 39 patients (27.1%) at 24 months after SDR. The mean increase between baseline and 24 months post-SDR was 2.4 units (95% CI 1.7–3.1, *p* < 0.001, annual change 1.2 units). Of the 67 patients with a GMFM-66 measurement available, a documented increase in gross motor function was seen in 77.6% (*n* = 52). Of 101 patients with spasticity data available, mean Ashworth scale decreased after surgery (2.74 to 0.30). Of patients’ pain scores, 60.7% (*n* = 34) improved, and 96.4% (*n* = 56) of patients’ pain scores remained the same or improved. Bladder function improved in 30.9% of patients.

**Conclusions:**

SDR improved gross motor function and reduced pain in most patients at 24 months after surgery, although the improvement is less pronounced than in children with GMFCS levels II and III. SDR should be considered in non-ambulant patients.

## Introduction

Cerebral palsy has a worldwide incidence of 2–3 per 1000 live births, of which 80% of children have spasticity in the lower limbs [1, 2]. Spasticity has a deleterious effect on motor function and quality of life [3]. Medical treatments available to treat spasticity include oral baclofen therapy, botulinum toxin, and intrathecal baclofen (ITB), as well as physiotherapy [4, 5].

Surgical management includes selective dorsal rhizotomy (SDR) a spinal procedure which involves the division of a proportion of the dorsal rootlets between L2 and S1. This reduces the sensory input into reflex arcs responsible for increased muscle tone, while preserving voluntary movement [6, 7]. Although current randomized control trial (RCT) evidence is limited [8], a recent multi-center, prospective observational study demonstrated significant improvement in gross motor function and quality of life scores in children 24 months after SDR [7, 9]. This cohort however was restricted to ambulatory children with a gross motor function classification system (GMFCS) level of II and III only [10].

There is limited evidence assessing the effectiveness of SDR in non-ambulatory children with cerebral palsy (GMFCS levels IV and V) [11]. This population is often excluded from randomized trials and observational studies, on the justification that they will not benefit as much from the procedure [9, 12, 13]. Currently, in the United Kingdom (UK), these children are not funded for SDR by the National Health Service (NHS). Smaller, single-center studies have suggested that these patients may also benefit from SDR [14, 15], although this has yet to be confirmed by larger, prospective studies, and the exact magnitude of the benefit obtained relative to ambulatory patients is also unclear. The effect on quality of life, motor, and spasticity outcomes has been indicated to be favorable [16], with an increase in gross motor function and spasticity, but there is a need for prospective evidence of this association [17].

## Objectives

The primary objective of this study was to conduct a multi-center prospective study to determine the effect of SDR on gross motor function (through assessment of the 66-item Gross Motor Function Measure [GMFM-66]) in non-ambulatory patients with spastic cerebral palsy (GMFCS levels IV and V). Motor function is associated with improved quality of life and overall function in CP patients [18]. In addition, we evaluated the feasibility of SDR in this patient group, by incorporating other reported outcome measures, specific to each center (including spasticity, pain, bladder function, and quality of life) and compared the changes in motor function with those previously reported for ambulant children with CP.

## Methods

### Study design

We carried out a prospective observational study in three NHS pediatric neurosurgical centers in England (Alder Hey Children’s Hospital NHS Trust [Liverpool]; Great Ormond Street Hospital NHS Trust [London], and Nottingham University Hospitals, [Nottingham]) of all non-ambulatory patients (GMFCS IV and V) with cerebral palsy, who underwent SDR between 2012 and 2019. Each center was experienced in delivering multi-modal treatments for spasticity, including botulinum toxin injections, ITB, selective peripheral neurectomy, and deep brain stimulation. Local audit approval at each unit was obtained prior to commencement of the study.

### Eligibility criteria

Eligible children had bilateral spastic cerebral palsy limiting functional capabilities, and were suitable candidates for surgery as determined by a multi-disciplinary (MDT) panel at each center. This panel included qualified (board certified) neurosurgeons, physiotherapists, occupational therapists, electrophysiologists, and neurosurgery nurses. Patients aged from 3 to 18 years, and operated within the time period, were eligible for the study. Patients were excluded if they could not attend follow-up appointments and community-based physiotherapy after SDR, due to finance, resource, or logistical reasons. All patients were deemed good candidates for surgery following MDT review, and were felt to be likely to benefit from the procedure. The eligibility process was designed to balance functional abilities and impairments and potential to benefit from surgery. No strict inclusion criteria were used across all centers, but patients were excluded if they had a progressive neurological condition or MRI confirmation of damage to key areas controlling posture and coordination, such as the basal ganglia or cerebellum. Patients with a GMFCS level I–III (ambulatory) at the time of surgery were excluded.

### Surgical technique

SDR was performed at all participating centers using a single-level laminectomy or laminoplasty approach, with neurophysiology-guided partial section of dorsal nerve roots. Intraoperative neurophysiology was used to guide the selection of the nerve rootlets. Each nerve root from L1 to S1 was divided into smaller portions of approximately equal size, followed by testing with neurophysiology to elicit a reflex motor response, recorded and graded for extent of spread to myotome levels. Rootlets that contributed to the greatest neurophysiology responses were cut as standard [9, 19].

### Treatment and follow-up

Specific post-operative protocols varied at each center, but patients were generally enrolled to a programme of post-operative physiotherapy lasting at least 24 months after surgery. Follow-up assessments were carried out at 3–6 monthly intervals depending on the center, up to a minimum of 24 months after surgery. All follow-up assessments were conducted by trained physiotherapists.

### Study outcomes

The primary outcome measure was the change in GMFM-66 (higher scores representing greater motor function) [20]. GMFM-66 has been shown to be a validated measure of gross motor function in patients with CP [21]. Spasticity was defined according to the Modified Ashworth Scale (MAS), with a mean calculated before and after SDR. Secondary outcomes were other measures collected by each participating center and included if parents/carers were “pleased, unsure, or not pleased” with the outcome of SDR at 24 months (1 center) and bladder function (improved, remained the same, or declined) (1 centerdefined as pre- and post-operative bladder continence assessments performed by a urologist with urodynamic studies, if available). Pain was reported by the patient, and scored on a scale of 1–10, with higher scores representing increasing pain severity. CPQoL scores were recorded from parent’s assessment. This included the pain reported component.

### Statistical analysis

Baseline demographics are summarized using descriptive statistics. Continuous variables were subject to a Kolmogorov–Smirnov test of normality-normally distributed variables are presented using the mean and standard deviation (SD) and skewed variables using the median and interquartile range (IQR). To assess longitudinal changes in GMFM-66 to follow individual patients’ trajectories, we used a linear mixed effects model, in which the patient was the random effect, with time after SDR, sex, and age as the fixed effect. A restricted log likelihood was determined to analyze the model with the best fit for each variable (AR [1], unstructured, compound symmetry, and diagonal), and differences over time were assessed by fitting an interaction term in the model with a likelihood ratio test. As previous studies have used 24 months after SDR as an appropriate time point, results were scaled to this with 95% confidence intervals (CIs) using a paired samples *t* test. Model fit and assumptions were examined through the use of residual plots. We assumed a *p* value of < 0.05 for statistical significance, with no corrections for multiple hypothesis testing. Spaghetti and smoothed conditional mean plots were used to represent longitudinal trends over time. Data analysis was conducted using R V4.02, and the figures were displayed using RStudio (ggplot2 package). Baseline differences in cohorts with missing outcomes at 24 months were compared using chi-squared tests.

## Results

### Patients and demographics

Between 2012 and 2019, 144 children underwent SDR in the three study centers. Sixty-seven patients did not have any GMFM-66 measurement follow-up data available, leaving 77 patients included in the analysis. Patient demographics are summarized in Table 1. The mean age at SDR was 8.2 years (SD 3.9). Most patients were female (52.1%, *n* = 75). Ninety-six patients were GMFCS level IV before surgery (66.7%), and 48 patients level V (33.3%). Of 90 patients with spasticity classification available, 87.8% had pure spasticity (*n* = 78/90). All patients had rootlets cut in L2-S1.

**Table 1:** Baseline characteristics

**Age at SDR (years)**	
**Mean (SD)**	**8.2 (0.3)**
Range	3–18
**Sex**	
Male	69/144 (47.9%)
Female	75/144 (52.1%)
**GMFCS level**	
IV	96/144 (66.7%)
V	48/144 (33.3%)
**Centers**	
Center 1	55/144 (38.2%)
Center 2	55/144 (38.2%)
Center 3	34/144 (23.6%)

### Scoring parameter changes

**Fig. 1:**
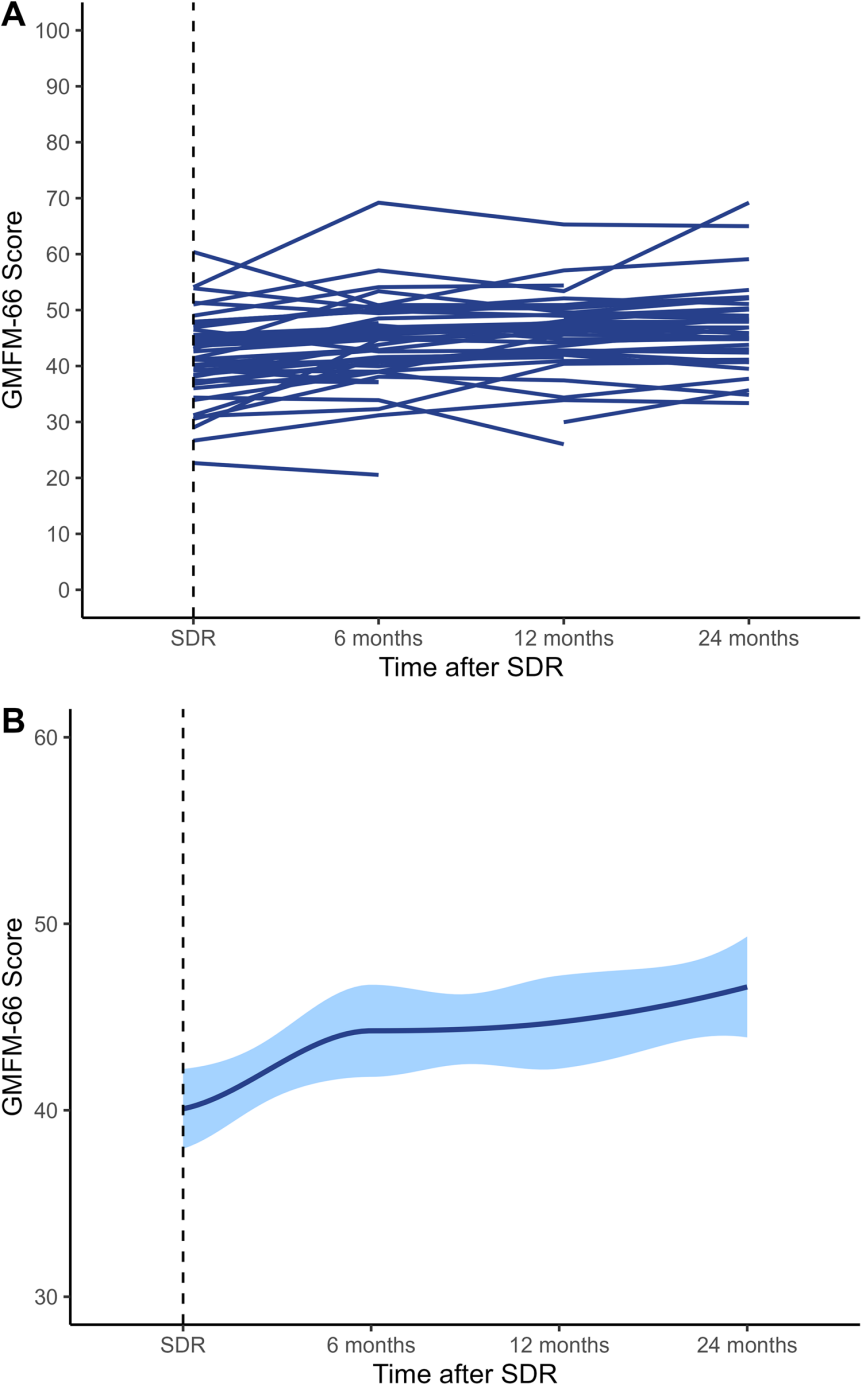
**A** Spaghetti plot of GMFM-66 scores over time after selective dorsal rhizotomy. **B** Smooth conditional mean plot with standard error over time (*y* ≠ 0)

**Fig. 2:**
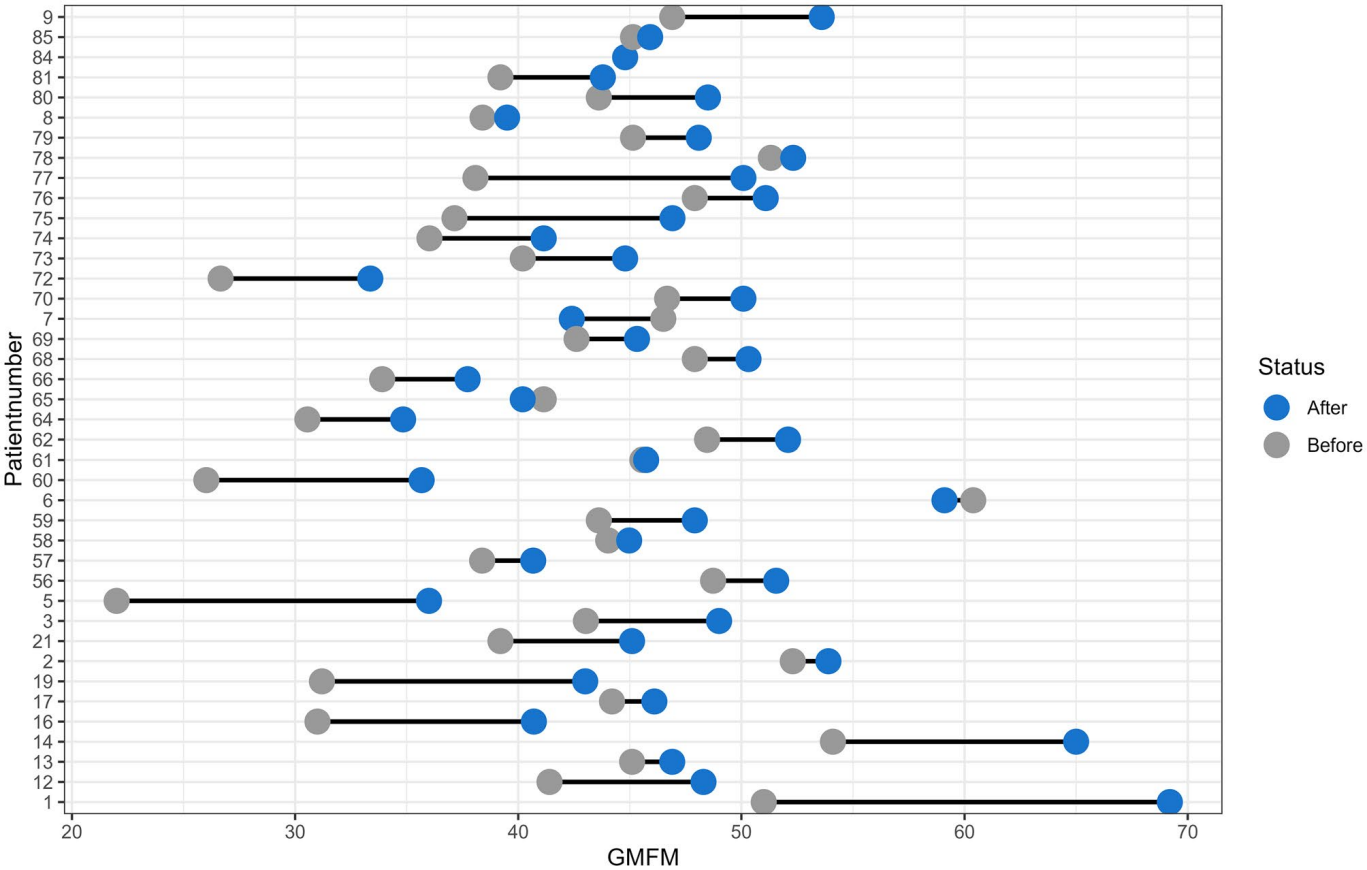
Dumbbell plot demonstrating individual change in GMFM-66 scores before and after SDR

**Fig. 3:**
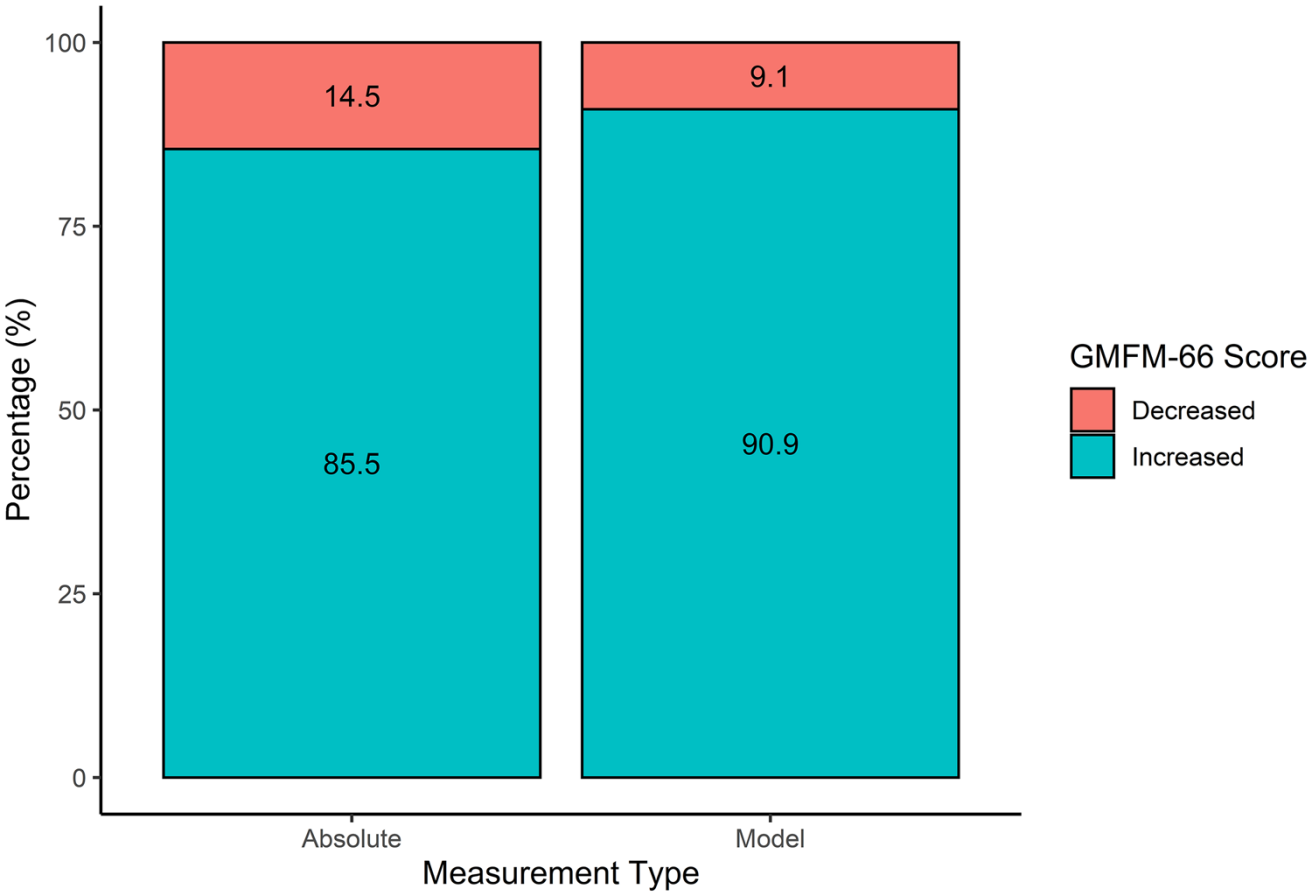
Stacked bar chart demonstrating improvements following SDR (green), and those that declined in GMFM-66 (red) in absolute terms, and according to the linear mixed effects model

**Fig. 4:**
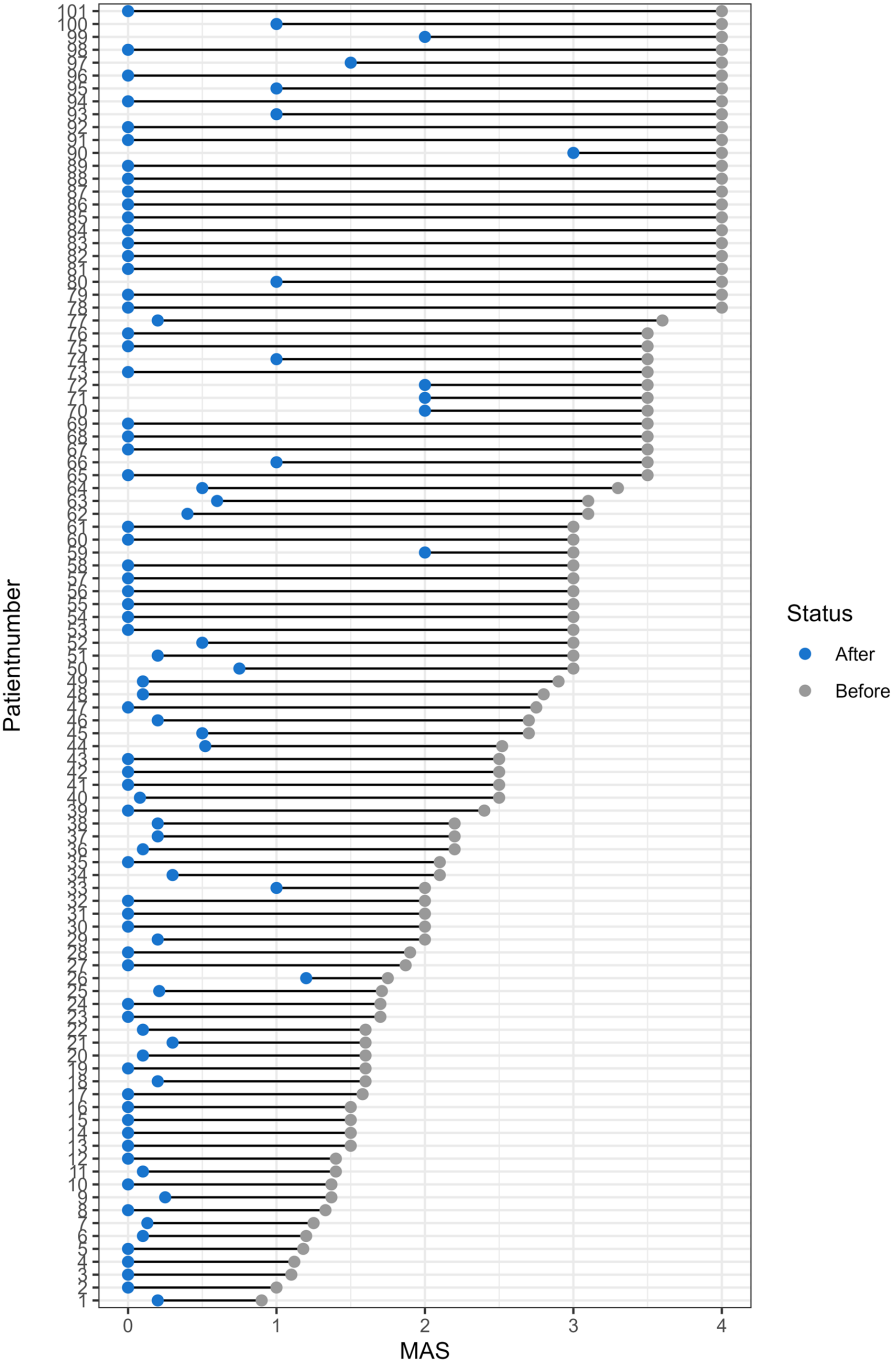
Dumbbell plot demonstrating individual change in MAS scores before and after SDR

The absolute GMFM-66 scores increased in most children from before surgery to 24 months after surgery (85.5%, *n* = 47/55) (Figs. 1 and 2), with the model demonstrating an increase in 90.9% (*n* = 70/77) (Fig. 3). The mean increase overall was 2.4 units (SD 2.8, 95% CI 1.7–3.1, *p* < 0.001), equating to a mean increase per year of 1.2 units (SD 1.4) (Table 2). The estimated increase was lower in patients with GMFCS level IV than in those with GMFCS level V; however, this was not significant (2.3 vs 5.2, *p* = 0.077). Spasticity changes are shown in Fig. 4 and Table 2. In total, 101 patients had spasticity scores available. There was a decrease in mean MAS scores after SDR (2.74 before to 0.30 after SDR) (Fig. 4).

**Table 2:** Primary outcomes

	**Before SDR**	**24 months after SDR**	**Modelled overall mean change (SD)**	**Modelled mean change per year (SD)**
**GMFM-66**				
**All patients**	40.20 (9.00)	42.61 (8.00)	2.40 (2.80)	1.20 (1.38)
**GMFCS level IV**	41.23 (7.44)	43.50 (6.74)	2.27 (2.72)	1.13 (1.36)
**GMFCS level V**	18.56 (13.00)	23.73 (10.08)	5.16 (2.92)	2.58 (1.46)
**MAS score**				
**All patients**	2.74 (1.00)	0.30 (0.58)	− 2.50 (1.00)	− 1.25 (0.50)
**GMFCS level IV**	2.28 (0.91)	0.22 (0.41)	− 2.10 (0.87)	− 1.03 (0.43)
**GMFCS level V**	3.49 (0.61)	0.42 (0.77)	− 3.06 (0.91)	− 1.53 (0.46)

### Follow-up

One hundred twenty-two (84.7%) patients had follow-up lasting 24 months. There were significant differences between the group with follow-up data available and the group without follow-up (Table 3). With the 55 patients with a parent/carer reported outcome at 24 months, 51 (92.7%) were “pleased” with the outcome, 2 (3.6%) were “not pleased”, and 2 (3.6%) were unsure. Bladder function outcomes were available for 55 patients at 24 months, of which 17 (30.9%) improved and 38 (69.1%) remained the same. Pain scores were available for 56 patients at 24 months, with 34 (60.7%) improving, 20 (35.7%) remaining the same, and 2 (3.6%) reporting increased pain. The mean improvement in 29 patients with CPQoL parent scores has been reported previously (mean increase 570 [95% CI 3175.0–3745.4], *p* < 0.001) [19].

**Table 3:** Comparisons with missing data cohort and regular cohort

**Parameter**	**Regular cohort**	**Missing cohort**	***p* value**
**Mean age (SD)**	9.5 (4.2)	6.5 (2.5)	< 0.001
**Female (%)**	57.1	48.9	0.332
**Baseline GMFM (SD)**	42.0 (7.4)	33.2 (15.0)	0.003
**Dystonia**	3.7	15.9	0.106
**Baseline GMFCS IV**	100	45.5	< 0.001

## Discussion

This prospective multi-center study showed that SDR led to a statistically significant increase in GMFM-66 scores and reduction in spasticity at 24 months. There was no difference between those children in GMFCS levels IV and V. However, this increase was less than those observed in previously reported studies in ambulant children [9, 22, 23]. This is in line with previous single-center studies examining SDR in non-ambulatory patients [15, 19]. Our study adds multi-center prospective evidence to suggest that SDR can be of benefit in improving motor function. The improvement was above what can be considered a minimal clinically important difference (MCID) in non-ambulatory populations [2]. Although quality of life is multifactorial in CP, and not solely based on pre-existing motor function, increased motor function may be associated with increased social participation and subsequent quality of life [24], and any improvement seen may be of benefit to patients and carers. A study examining electrophysiology findings in non-ambulatory children with CP compared to controls at surgery identified that SDR decreased isometric muscle activity by almost 40%, and post-operatively reduced the difference in muscle activity compared to the control group by 50% [25]. This indicates SDR may lead to motor improvements through the mechanism of decreasing isometric and overall muscle activity.

The smaller increase compared to that exhibited in ambulatory patients is in congruity with a case–control analysis, which demonstrated that changes in GMFM scores decreased with increasing GMFCS level [16]. Furthermore, a long-term outcome study assessing SDR identified that the benefit experienced by ambulatory children was not observed in those with GMFCS levels IV and V [26]. Nonetheless, SDR was noted to lead to improvement in spasticity and urological dysfunction that was sustained at follow-up in GMFCS IV and V patients, leading to a reduction in spasticity [15].

A recent study has suggested that the benefits of SDR relating to quality of life, and muscle strength, are maintained after SDR [27]. Another study suggested that the benefit of SDR in GMFCS II and III patients increased between 12 and 24 months, with a greater degree of improvement seen [17, 19]. Even if SDR is not successful by measured criteria, the vast majority of patients still report improved functioning, even if this was not quantifiable [28].

A published systematic review, examining the outcomes of ITB and SDR in non-ambulant populations specifically, suggested that ITB had a significantly higher complication rate than SDR [11]. Of twenty-seven studies, most were case series, and the authors concluded that larger, prospective multi-center studies were required in order to confirm the utility of both treatment modalities in the population. We aimed to provide this with this study, using the defined outcome measure of GMFM-66.

Our findings must be interpreted in the context of the CP disease course. Younger patients under the age of 5 years will have a degree of natural increase in GMFM and an evolving clinical course, which must be considered for the population < 5 years included in our study. In addition, the patients with missing GMFM data in the cohort are significantly younger and had significantly lower GMFM scores and GMFCS level, than those with follow-up data available. The results of this study therefore do not fully reflect this population, and efforts will need to be made to understand reasons behind the attrition seen in these groups.

There is evidence that SDR improves both bladder storage characteristics and overall function [29]. Our results support these findings and indicate that most GMFCS level IV and V patients that undergo SDR either improve or have the same function after surgery, possibly leading to improved quality of life beyond increased motor function [30].

### Strengths of the study

This study has several strengths. First, it is a multi-center study of prospectively enrolled patients with GMFCS levels IV and V undergoing SDR at three pediatric neurosurgical centers, with similar surgical techniques and follow-up protocols.

Second, to the author’s knowledge, this study is the largest reported sample of patients with GMFCS levels IV and V undergoing SDR, with a large number of both levels represented in this study. Third, we used the most widely validated and accepted tool to measure gross motor function (GMFM-66). Fourth, we utilized a linear mixed effects model to account for missing values and irregular follow-up times and facilitate inclusion of every patient in the study with a GMFM measurement available.

### Limitations of the study

The study has several limitations. First, one center did not collect GMFM-66 measurements as part of their standardized follow-up protocol, and therefore, we were unable to include them in the analysis. Furthermore, while each center carried out their own specific tests, there was little overlap in these, and therefore, the results are not reflective of all patients that are included in this study. The bladder outcomes, in particular, were also not objective, but reported according to each center’s own criteria. The findings of this outcome therefore should not be taken as definitive evidence that SDR improves bladder function in this group. We did also not include details of post-operative complications, which are an important component of evaluating the success of SDRalthough we aimed to evaluate long-term functional outcomes as opposed to immediate complications.

Furthermore, the longitudinal outcomes did not examine the presence of worsening or new spinal deformities as a result of SDRthis is estimated to affect up to 50% of children with CP at some stage [31], with SDR worsening this in up to a third of cases [32]. This association, and the ways of monitoring children for spinal deformities, will need to be examined in future studies. It is important that in some studies, this has not led to any significant clinical deficits [33], but the likelihood of requiring further intervention for this should be considered in these populations, especially after undergoing SDR.

Third, although this study contains data from multiple centers, this is limited to neurosurgery units located in the UK, and therefore, the results may not be generalizable to international populations. Multi-center studies have reported significant differences in the way spastic diplegic CP is managed between European countries, with differing uses of botulinum toxin, intrathecal baclofen, and selective dorsal rhizotomy all exhibited [34]. This may reflect differential accessibility of countries and units to SDR, and given the benefits of the procedure, SDR should be available to all tertiary pediatric neurosurgical units globally when possible.

### Implications for practice

Our results have several implications. First, they indicate that SDR is plausible in non-ambulatory populations, and doing so generally leads to an increase in motor function. This could be considered by policy makers and healthcare service provision providers. In the UK, the NHS is responsible for commission of SDR, and currently, the procedure is funded for patients with GMFCS levels II and III. This study suggests that patients with GMFCS levels IV and V may benefit additionally from SDR.

Furthermore, the eligibility criteria for SDR is often questioned and limited to ambulatory patientsour results show that the benefits, while not as significant improvement as seen in ambulatory patients, still demonstrate a motor improvement. This should be considered by multi-disciplinary teams when assessing eligibility for SDR. SDR and its effects are often portrayed favorably by the media [35], and there is a need to remain pragmatic to manage this expectation effectively among parents and caregivers of children affected. Despite the aforementioned benefits, SDR still requires regular post-operative physiotherapy and multi-disciplinary team support and remains a labor-intensive treatment that carries a risk of complications [36].

## Conclusions

This multi-center prospective study demonstrates evidence that the improvements seen after SDR are also exhibited in non-ambulatory populations. The observed study supports that SDR is beneficial in patients with GMFCS levels IV and V; however, ultimately, this is less beneficial than in traditional candidates for the procedure.

## Data Availability

Anonymized data are available on reasonable request, by contacting the corresponding author.

## Declarations

### Copyright

The authors declare that the work submitted is their own and that copyright has not been breached in seeking its publication.
